# Gut microbiota and metabolomic characteristics associated with metabolic syndrome in post-cholecystectomy patients: a retrospective cross-sectional study

**DOI:** 10.3389/fmed.2026.1849889

**Published:** 2026-06-22

**Authors:** Yue Lou, Xiangping Wang

**Affiliations:** 1 Hepatobiliary and Pancreatic Surgery, Shaoxing Second Hospital, Yuecheng, Shaoxing, China; 2 Department of Hematology, Shaoxing Second Hospital, Yuecheng, Shaoxing, China

**Keywords:** bile acids, cholecystectomy, gut microbiota, metabolic syndrome, short-chain fatty acids

## Abstract

**Background:**

Cholecystectomy (CCE) is widely performed for gallbladder disease, and postoperative bile acid metabolism and gut microbiota homeostasis have attracted increasing attention. Patients with metabolic syndrome (MS) may show distinct postoperative microbiota and metabolomic features, yet these characteristics remain poorly concerned in post-CCE populations. This study compared gut microbiota and metabolic profiles between post-CCE patients with and without MS.

**Methods:**

A retrospective cross-sectional study of 432 cases was analyzed. Fecal samples were subjected to 16S rRNA gene sequencing and metabolomic profiling. Microbial diversity, taxonomic composition, functional pathways, and metabolite levels were compared between groups and integrated with clinical parameters.

**Results:**

MS patients exhibited reduced microbial richness, reflected by lower Chao1 richness, while Shannon and Simpson indices were not significantly different. The MS group also showed a lower Firmicutes/Bacteroidetes ratio, increased Prevotella abundance, and reduced Bacteroides, Faecalibacterium, and Roseburia abundance. Metabolomic analysis revealed elevated secondary bile acids and diminished short-chain fatty acids, particularly butyrate. Correlation analysis linked these changes to inflammatory markers and adverse metabolic indices. Functional prediction analysis using PICRUSt2 suggested potential enrichment of bile acid metabolism and pro-inflammatory signaling pathways in the MS group.

**Conclusions:**

In conclusion, among patients with a history of CCE, those with MS exhibited distinct gut microbial and metabolomic profiles compared with non-MS patients, and these profiles were associated with inflammatory and metabolic indicators. These findings suggest that dietary modulation, probiotic supplementation, and other microbiota-targeted strategies should be investigated in future prospective interventional studies rather than interpreted as established therapeutic approaches.

## Introduction

Cholecystectomy (CCE) is one of the most common surgical procedures worldwide and is the preferred treatment for symptomatic gallstones, chronic cholecystitis, and gallbladder polyps. However, distinguishing the independent effect of CCE from pre-existing metabolic differences requires controlled preoperative and postoperative comparisons. Patients frequently develop long-term complications such as post-cholecystectomy diarrhea (PCD), dyslipidemia, non-alcoholic fatty liver disease (NAFLD), and even colorectal cancer (CRC) (1–4), which is of particular concern in individuals with metabolic syndrome (MS), who already carry a heavy burden of metabolic and cardiovascular risk (5).

The gallbladder plays an essential role in regulating bile acid (BA) flow. By storing and concentrating bile, it ensures rhythmic BA release after meals. Following CCE, continuous BA secretion into the intestine disrupts enterohepatic circulation and alters luminal BA composition (6, 7), increasing exposure to primary BAs and enhancing microbial transformation into secondary BAs such as deoxycholic acid (DCA) and lithocholic acid (LCA). Elevated secondary BAs exert cytotoxic and pro-inflammatory effects, while simultaneously signaling through FXR and TGR5 pathways to affect glucose, lipid, and energy metabolism (8–10). Such BA-microbiota crosstalk provides a mechanistic basis for the metabolic disturbances observed after CCE. Recent studies further suggest that CCE may profoundly alter gut microbiota composition. Multiple cross-sectional and cohort analyses show enrichment of bile-acid–transforming bacteria such as *Bilophila, Bacteroides*, and *Megamonas*, and depletion of short-chain fatty acid (SCFA) producers including *Faecalibacterium* and *Roseburia* (11, 12). Xu et al. (1) demonstrated that CCE significantly disrupts gut microbiota homeostasis and reduces fecal SCFA levels, and Yoon et al. (3) reported decreased microbial diversity and enrichment of Blautia obeum and Veillonella parvula after CCE. In addition, studies in animal models confirm that BA disturbances drive blooms of pathobionts such as *Bilophila wadsworthia*, which in turn promote inflammation (13).

MS, characterized by central obesity, hypertension, hyperglycemia, hypertriglyceridemia, and reduced HDL cholesterol, is highly prevalent worldwide, affecting 20–45% of adults (5, 14). It predisposes to type 2 diabetes, NAFLD, and cardiovascular disease. Importantly, the chronic inflammation, insulin resistance, and dyslipidemia inherent in MS may coexist with more pronounced gut microbiota and bile acid disturbances in patients after CCE (5, 15). Yu et al. (15) recently showed that MS patients undergoing laparoscopic CCE exhibit distinct intraoperative metabolic vulnerabilities compared with non-MS patients, suggesting an amplified physiological burden. Furthermore, dietary factors may further shape these interactions, for instance, high-fat diets, particularly those rich in milk-derived saturated fat, promote taurine-conjugated BA production and expansion of sulfite-reducing bacteria like *Bilophila*, driving colitis in susceptible hosts (13, 16). Conversely, dietary fiber enhances SCFA production and supports intestinal homeostasis (17). Previous studies suggest that high-fat and high-cholesterol dietary patterns may be associated with more pronounced microbial and metabolic disturbances after CCE, including increased intestinal inflammation (1, 4). The small intestine microbiota also regulates lipid digestion and absorption, thereby mediating systemic responses to dietary fat (18). Thus, diet emerges as both a risk factor and a potential therapeutic target in post-CCE patients with MS.

Microbiota and metabolomic alterations observed in post-CCE populations may have clinical relevance, although their causal relationship with CCE remains incompletely established. Elevated secondary BAs have been linked with increased CRC risk (19). Dysbiosis characterized by loss of SCFA-producing taxa impairs gut barrier integrity and promotes systemic inflammation, contributing to NAFLD and cardiometabolic diseases (20, 21). Furthermore, PCD, which affects up to 57% of patients, is increasingly recognized as microbiota-driven. Xu et al. (2) demonstrated that altered gut microbiota after CCE promotes secondary bile acid accumulation, which stimulates serotonin production and accelerates colonic motility. Despite these insights, most existing studies are limited by small cohorts or cross-sectional designs. Large-scale studies comparing gut microbiota and metabolomic profiles among post-CCE patients with different metabolic statuses remain limited. Few studies have integrated microbiome sequencing with bile acid and SCFA profiling in large clinical populations (1, 4). Therefore, comprehensive retrospective studies are needed to characterize these microbiota and metabolomic alterations and clarify their clinical significance.

In the current study, we retrospectively analyzed 432 patients who had undergone CCE at least 12 months previously, including patients with and without MS. We aimed to compare gut microbiota diversity, taxonomic composition, bile acid metabolism, and SCFA profiles between post-CCE patients with MS and those without MS. By integrating microbiome and metabolomic analyses, this study sought to clarify the microbiota–metabolite features associated with MS status in a post-CCE population, rather than to infer longitudinal effects of CCE.

## Methods

### Study design and participants

This retrospective cross-sectional association study included 432 patients who had undergone laparoscopic cholecystectomy between June 2022 and December 2024 at Shaoxing Second Hospital and had postoperative clinical, fecal microbiota, and metabolomic data available. Because fecal samples were collected only after CCE and no preoperative microbiota or metabolomic data were available, the study was designed to compare postoperative MS and non-MS patients rather than to assess longitudinal changes caused by CCE. Preoperative gallbladder disease patients who had not yet undergone CCE were not included; therefore, within-group pre–post comparisons in MS or non-MS patients and between-group comparisons of change magnitude could not be performed. Among them, 256 patients met the diagnostic criteria for metabolic syndrome (MS group) according to the International Diabetes Federation (IDF) guidelines, while 176 age- and sex-matched individuals without metabolic syndrome served as the non-MS group. Inclusion criteria were: (1) adults aged 18–70 years; (2) laparoscopic cholecystectomy performed at least 12 months prior; and (3) complete clinical and laboratory records. Exclusion criteria were: (1) history of inflammatory bowel disease, irritable bowel syndrome, gastrointestinal surgery other than cholecystectomy, or malignancy; (2) antibiotic, probiotic, or prebiotic use within 3 months; and (3) severe systemic diseases such as hepatic failure, renal failure, or autoimmune disorders. The study was approved by the institutional ethics committees (No. 2022069821A) of Shaoxing Second Hospital. Because this study used existing clinical records and anonymized laboratory data, the requirement for written informed consent was waived by the ethics committee. All procedures were conducted in accordance with the Declaration of Helsinki.

### Clinical and anthropometric assessment

Demographic and lifestyle data, including age, sex, body mass index (BMI), smoking and alcohol history, and dietary habits, were retrieved from hospital records and structured questionnaires. Blood pressure (BP) was measured twice using an automated sphygmomanometer (Omron HEM-907XL) after 10 min of seated rest, and the mean value was recorded. Waist circumference was measured with a non-stretchable tape at the midpoint between the lower margin of the last palpable rib and the top of the iliac crest at the end of normal expiration, according to the WHO standardized protocol (22). Clinical history, medication use, and metabolic syndrome components (obesity, dyslipidemia, hypertension, hyperglycemia) were collected for classification and subgroup analyses.

### Biochemical and metabolic parameters

Venous blood samples were collected after overnight fasting (≥10 h). Fasting blood glucose (FBG) and glycated hemoglobin (HbA1c) were determined using enzymatic assays and high-performance liquid chromatography (HPLC). Serum lipids, including triglycerides (TG), total cholesterol (TC), high-density lipoprotein cholesterol (HDL-C), and low-density lipoprotein cholesterol (LDL-C), were measured enzymatically on an automated biochemistry analyzer (Hitachi 7600). Liver function tests (ALT, AST, GGT, ALP, and bilirubin) and renal function (creatinine, urea nitrogen) were performed using standardized clinical chemistry methods. High-sensitivity C-reactive protein (hs-CRP) and fasting insulin were measured by immunoturbidimetry and chemiluminescence assays, respectively, and insulin resistance was estimated by the homeostasis model assessment (HOMA-IR). Oxidative stress biomarkers, including malondialdehyde (MDA) and superoxide dismutase (SOD), were quantified using spectrophotometric methods. Fecal bile acid profiles were analyzed using ultra-performance liquid chromatography coupled with tandem mass spectrometry (UPLC–MS/MS), quantifying both primary and secondary bile acids [cholic acid (CA), chenodeoxycholic acid (CDCA), deoxycholic acid (DCA), lithocholic acid (LCA), ursodeoxycholic acid (UDCA), and conjugated derivatives].

### Fecal sample collection and microbiota analysis

Participants provided fresh stool samples, which were collected in sterile containers, immediately frozen at −80 °C, and processed within one week. Microbial DNA was extracted using the QIAamp DNA Stool Mini Kit (Qiagen), following manufacturer instructions with bead-beating steps to ensure lysis of Gram-positive bacteria. The V3–V4 region of the 16S rRNA gene was amplified with primers 341F/806R, and sequencing was performed on an Illumina MiSeq platform (2 × 300 bp paired-end reads). Quality filtering, denoising, chimera removal, and amplicon sequence variant (ASV) generation were performed using the DADA2 pipeline in QIIME2 (23, 24). Taxonomic assignment was performed against the SILVA 132 database. The average sequencing depth was approximately 52,000 reads per sample after quality control. Alpha-diversity indices, including Shannon, Simpson, and Chao1 richness, and beta-diversity indices, including Bray–Curtis and weighted UniFrac distances, were calculated. Functional prediction was carried out using PICRUSt2 to infer microbial gene content from 16S rRNA sequencing data, and predicted functional profiles were annotated using the Kyoto Encyclopedia of Genes and Genomes (KEGG) pathway database (25, 26). To reduce the influence of rare or unstable taxa, ASVs/taxa with a relative abundance < 0.01% or present in < 10% of samples were excluded before genus-level comparative analyses. In addition, targeted quantification of selected bacterial taxa (*Bacteroides, Prevotella, Faecalibacterium, Roseburia, Bifidobacterium*, and *Blautia*) was performed by quantitative real-time PCR with species-specific primers.

### Fecal metabolomics and short-chain fatty acids

Untargeted fecal metabolomics was performed using ultra-performance liquid chromatography coupled with tandem mass spectrometry (UPLC–MS/MS; Waters ACQUITY system) operating in both positive and negative ion modes. Fecal samples were homogenized and extracted using methanol-based extraction solvents according to standard metabolomics protocols. Pooled quality control (QC) samples were prepared by mixing equal aliquots from all fecal extracts and were analyzed periodically throughout the analytical sequence to monitor instrument stability, signal reproducibility, and data consistency. Internal standards were added to each sample prior to extraction for normalization and quality assurance purposes. Raw metabolomics data were processed using standard peak extraction, retention-time alignment, peak integration, and normalization workflows. Metabolite identification was performed by matching accurate mass spectra and fragmentation patterns against the Human Metabolome Database (HMDB) and reference spectral libraries (27). Features with poor analytical reproducibility or excessive missing values were excluded during preprocessing, including features with a coefficient of variation (CV) >30% in pooled QC samples or missing values in >50% of all samples or >50% of samples within either the MS or non-MS group. For retained metabolites, remaining missing values were imputed using one-half of the minimum detected value for the corresponding metabolite. Batch effects and signal drift were corrected prior to downstream statistical analyses. Fecal bile acid profiles, including primary and secondary bile acids (CA, CDCA, DCA, LCA, UDCA, and conjugated derivatives), were analyzed using UPLC–MS/MS. Short-chain fatty acids (SCFAs), including acetate, propionate, butyrate, valerate, isobutyrate, and isovalerate, were quantified using gas chromatography–mass spectrometry (GC–MS) following derivatization with N-tert-butyldimethylsilyl-N-methyltrifluoroacetamide. SCFA concentrations were expressed as mmol/kg of wet feces.

### Lifestyle assessment

Smoking status, alcohol consumption, self-reported physical activity, and general dietary habits were retrieved from hospital records and structured clinical questionnaires when available. Because detailed quantitative dietary intake data, including validated food frequency questionnaire-derived macronutrient intake, cholesterol intake, fiber intake, and standardized International Physical Activity Questionnaire scores, were not consistently available in this retrospective cohort, lifestyle variables were mainly used for descriptive baseline comparison. Available categorical lifestyle variables, including smoking, alcohol use, and general dietary pattern, were also included as covariates in the multivariable-adjusted models.

### Statistical analysis

Data were analyzed using SPSS v26.0 and R software v4.3. Continuous variables were expressed as mean ± standard deviation or median with interquartile range depending on distribution, and categorical variables were expressed as numbers and percentages. Between-group comparisons were performed using Student's *t*-test or Welch's *t*-test for continuous variables and the chi-square test or Fisher's exact test for categorical variables, as appropriate.

To address potential confounding, both univariate and multivariable regression analyses were performed for key microbiota and metabolomic outcomes. In the univariate models, each microbiota or metabolomic feature was modeled using MS status as the only independent variable. In the multivariable models, MS status was entered as the primary exposure variable, with non-MS patients as the reference group. The models were adjusted for age, sex, BMI, smoking status, alcohol use, dietary pattern, antidiabetic medication, lipid-lowering medication, antihypertensive medication, bile acid-related medication, and time since cholecystectomy. Adjusted β coefficients, 95% confidence intervals, adjusted *p* values, and false discovery rate-adjusted q values were reported. False discovery rate correction was applied separately to univariate and multivariable analyses to account for multiple comparisons. A two-sided *p* value < 0.05 was considered statistically significant.

## Results

### Baseline characteristics and clinical parameters

**Figure 1:**
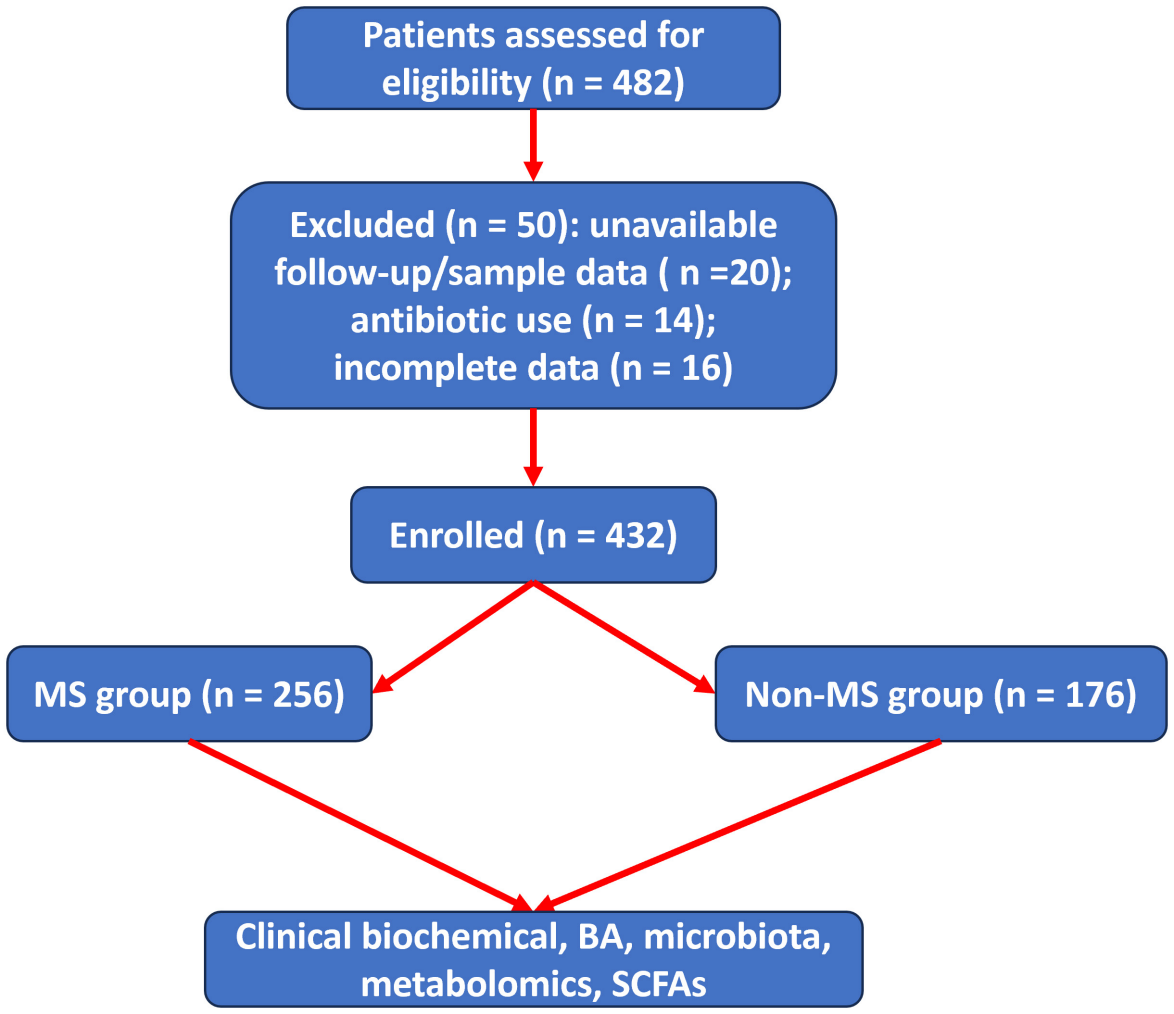
Study flowchart. Flow diagram of patient recruitment and grouping: 432 cases included, stratified into metabolic syndrome (MS) and non-MS after cholecystectomy, with exclusions detailed.

A total of 432 post-cholecystectomy patients were included, of whom 256 were diagnosed with metabolic syndrome (MS group) and 176 were classified as non-metabolic syndrome patients (non-MS group) (Figure 1). As summarized in Table 1, there were no significant differences between groups in mean age (58.4 ± 6.0 vs. 57.9 ± 6.4 years, *p* = 0.36) or sex distribution [121 (47.3%) vs. 80 (45.5%) male, *p* = 0.72]. Smoking status, alcohol consumption, and self-reported physical activity were broadly comparable between groups; however, detailed quantitative dietary intake and standardized physical activity data were not consistently available, and residual lifestyle-related confounding cannot be excluded. The MS group showed a distinctly unfavorable metabolic profile. BMI and waist circumference were significantly higher in the MS group than in the non-MS group (28.1 ± 3.2 vs. 26.4 ± 3.1 kg/m^2^ and 97.3 ± 9.1 vs. 91.6 ± 8.0 cm, respectively; both *p* < 0.01) (Table 1). Similarly, systolic blood pressure (SBP) and diastolic blood pressure (DBP) were elevated in the MS group (136.8 ± 14.2 vs. 131.2 ± 13.3 mmHg and 87.3 ± 9.3 vs. 83.5 ± 8.3 mmHg, respectively) (Table 1). Biochemical indicators reflected significant derangements in glucose and lipid metabolism. The MS group exhibited markedly higher fasting blood glucose (FBG) and glycated hemoglobin (HbA1c) levels (6.90 ± 1.31 vs. 5.70 ± 0.84 mmol/L and 6.80 ± 0.89 vs. 5.90 ± 0.67%, respectively; both *p* < 0.001) (Table 1). Serum triglycerides (TG), total cholesterol (TC), and low-density lipoprotein cholesterol (LDL-C) were higher in the MS group, whereas high-density lipoprotein cholesterol (HDL-C) was significantly lower, consistent with the dyslipidemic phenotype characteristic of MS (Table 1). Liver function tests showed elevated alanine aminotransferase (ALT), suggesting subtle hepatic injury or steatosis, whereas renal function, reflected by creatinine, was comparable between groups. Additional baseline variables relevant to potential confounding were also compared. The MS group had a higher proportion of patients with a high-fat/high-cholesterol dietary pattern and more frequent use of antidiabetic, lipid-lowering, and antihypertensive medications, while time since CCE and bile acid-related medication use were comparable between groups. These variables were therefore considered in subsequent multivariable-adjusted models.

**Table 1:** Baseline characteristics of post-CCE patients with and without MS.

Parameter	MS group (*n* = 256)	Non-MS group (*n* = 176)	*p*-value
Age (years)	58.4 ± 6.0	57.9 ± 6.4	0.36
Male (%)	121 (47.3)	80 (45.5)	0.72
BMI (kg/m^2^)	28.1 ± 3.2	26.4 ± 3.1	<0.01
Waist circumference (cm)	97.3 ± 9.1	91.6 ± 8.0	<0.01
SBP (mmHg)	136.8 ± 14.2	131.2 ± 13.3	<0.01
DBP (mmHg)	87.3 ± 9.3	83.5 ± 8.3	0.02
Smoking (%)	88 (34.4)	54 (30.7)	0.41
Alcohol use (%)	107 (41.8)	69 (39.2)	0.55
Physical activity (moderate/high %)	133 (52.0)	98 (55.7)	0.48
High-fat/high-cholesterol dietary pattern (%)	148 (57.8)	69 (39.2)	<0.001
Time since CCE (months)	24.9 ± 8.2	25.5 ± 7.4	0.41
Antidiabetic medication (%)	123 (48.0)	21 (11.9)	<0.001
Lipid-lowering medication (%)	108 (42.2)	32 (18.2)	<0.001
Antihypertensive medication (%)	141 (55.1)	42 (23.9)	<0.001
Bile acid-related medication (%)	20 (7.8)	11 (6.2)	0.67
FBG (mmol/L)	6.90 ± 1.31	5.70 ± 0.84	<0.001
HbA1c (%)	6.80 ± 0.89	5.90 ± 0.67	<0.001
TG (mmol/L)	2.10 ± 0.74	1.60 ± 0.42	<0.001
TC (mmol/L)	5.40 ± 0.80	4.80 ± 0.76	<0.01
HDL-C (mmol/L)	0.92 ± 0.22	1.21 ± 0.25	<0.001
LDL-C (mmol/L)	3.20 ± 0.74	2.70 ± 0.65	<0.01
ALT (U/L)	42.7 ± 13.3	34.5 ± 11.6	<0.01
Creatinine (μmol/L)	76.3 ± 13.2	73.5 ± 13.0	0.29

### Inflammatory, insulin resistance, and oxidative stress markers

Systemic inflammatory and oxidative stress markers, as well as indicators of insulin resistance and endothelial activation were also detected. The MS group exhibited a heightened inflammatory burden, with hs-CRP levels nearly doubled compared to non-MS patients (6.3 ± 2.2 vs. 3.8 ± 1.6 mg/L, *p* < 0.001) (Table 2). Markers of insulin resistance were markedly elevated. Fasting insulin levels were nearly twice as high in the MS group (18.7 ± 6.6 vs. 10.4 ± 3.6 μU/mL, *p* < 0.001), and HOMA-IR values confirmed severe insulin resistance (5.7 ± 2.1 vs. 2.8 ± 1.0, *p* < 0.001) (Table 2). This aligns with the observed hyperglycemia and dyslipidemia, suggesting integrated metabolic–immune dysfunction.

**Table 2:** Inflammatory, insulin resistance, and oxidative stress markers in post-CCE patients with and without MS.

Marker	MS group (*n* = 256)	Non-MS group (*n* = 176)	*p*-value
hs-CRP (mg/L)	6.3 ± 2.2	3.8 ± 1.6	<0.001
Insulin (μU/mL)	18.7 ± 6.6	10.4 ± 3.6	<0.001
HOMA-IR	5.7 ± 2.1	2.8 ± 1.0	<0.001
MDA (nmol/mL)	5.2 ± 1.3	3.6 ± 1.1	<0.001
SOD (U/mL)	85.4 ± 15.3	101.7 ± 17.6	<0.001
ICAM-1 (ng/mL)	182.4 ± 32.7	148.9 ± 28.1	<0.001
VCAM-1 (ng/mL)	394.5 ± 71.2	318.2 ± 66.3	<0.001

Oxidative stress was significantly more pronounced in MS patients. Malondialdehyde (MDA), a marker of lipid peroxidation, was elevated (5.2 ± 1.3 vs. 3.6 ± 1.1 nmol/mL, *p* < 0.001), while antioxidant enzyme activity, measured by superoxide dismutase (SOD), was suppressed (85.4 ± 15.3 vs. 101.7 ± 17.6 U/mL, *p* < 0.001) (Table 2). These imbalances indicate excessive oxidative damage and insufficient defense mechanisms in MS patients.

Endothelial activation was reflected by significantly higher serum levels of ICAM-1 and VCAM-1 in the MS group (182.4 ± 32.7 vs. 148.9 ± 28.1 ng/mL and 394.5 ± 71.2 vs. 318.2 ± 66.3 ng/mL, respectively, both *p* < 0.001) (Table 2). Taken together, these results demonstrate that patients with MS after cholecystectomy exhibit not only worsened metabolic indices but also systemic inflammation, oxidative stress, and endothelial dysfunction, creating a high-risk cardiovascular and metabolic phenotype.

### Gut microbiota diversity, composition, and bile acid/SCFA profiles

**Figure 2:**
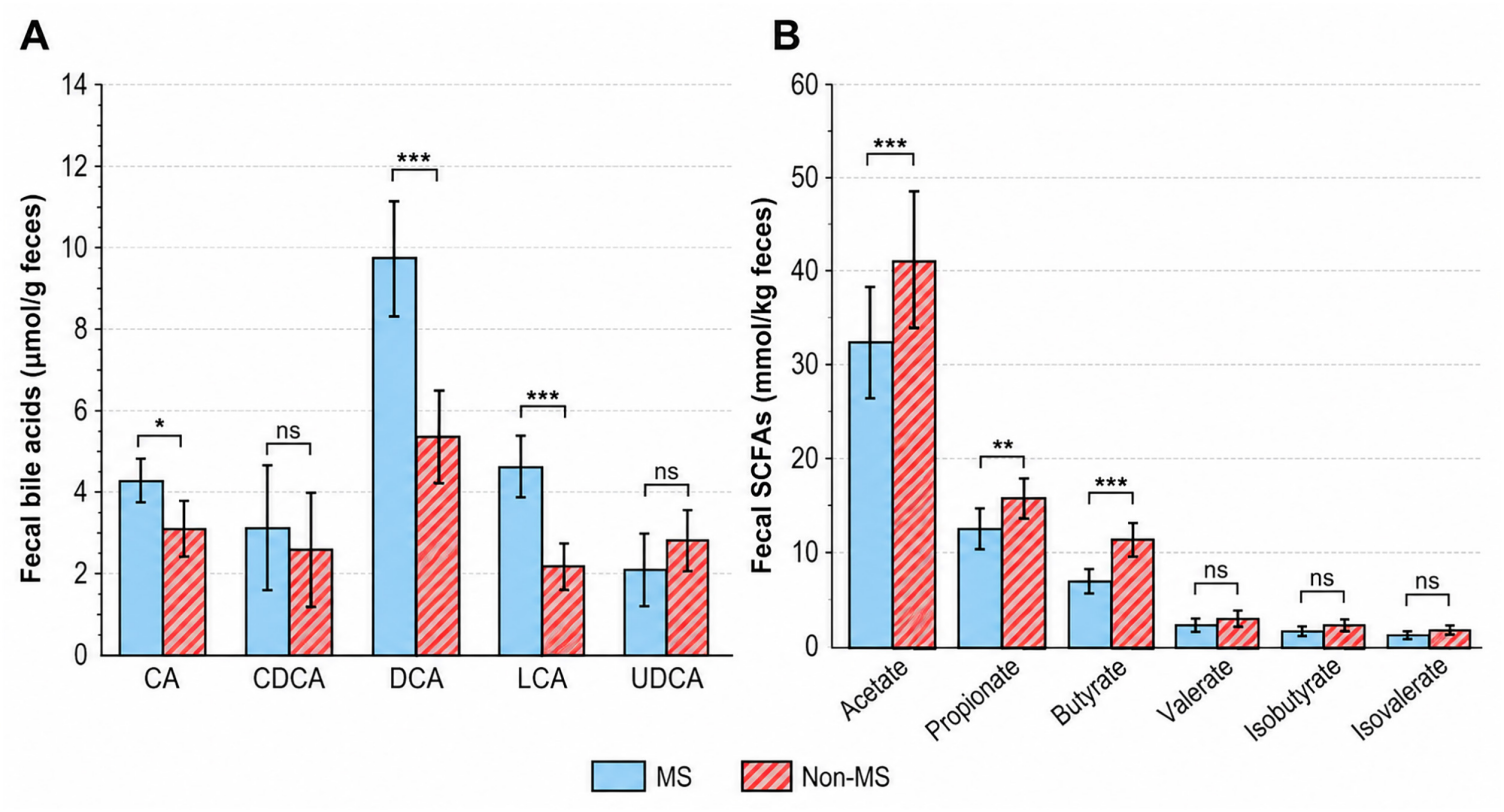
Differences regarding fecal bile acid and short-chain fatty acid concentrations in post-CCE patients with and without MS. **(A)** Fecal bile acid concentrations, including CA, CDCA, DCA, LCA, and UDCA. **(B)** Fecal short-chain fatty acid concentrations, including acetate, propionate, butyrate, valerate, isobutyrate, and isovalerate. *p < 0.05, **p < 0.01, ***p < 0.001; ns, not significant. CCE, cholecystectomy; MS, metabolic syndrome; CA, cholic acid; CDCA, chenodeoxycholic acid; DCA, deoxycholic acid; LCA, lithocholic acid; UDCA, ursodeoxycholic acid; SCFAs, short-chain fatty acids.

All microbiota and metabolomic comparisons were based on postoperative samples, and therefore, the results reflect cross-sectional differences between post-CCE MS and non-MS patients rather than pre–post changes after surgery. Bile acid profiling revealed distinct fecal bile acid differences between post-CCE patients with and without MS. In Figure 2A, the MS group is presented as the first/left bar and the non-MS group as the second/right bar for each metabolite. Compared with non-MS patients, MS patients showed significantly higher fecal CA, DCA, and LCA concentrations, whereas CDCA and UDCA did not differ significantly between groups. The most pronounced differences were observed for the secondary bile acids DCA and LCA, suggesting that MS status in post-CCE patients was associated with a more unfavorable bile acid profile. Fecal SCFA concentrations are shown in Figure 2B, with the MS group consistently presented as the first/left bar and the non-MS group as the second/right bar. Acetate, propionate, and butyrate concentrations were significantly lower in the MS group than in the non-MS group, whereas valerate, isobutyrate, and isovalerate showed no significant between-group differences. These findings indicate that post-CCE patients with MS had reduced levels of major beneficial SCFAs, particularly butyrate, while minor branched-chain SCFAs were not significantly altered. Together, these data indicate that, among post-CCE patients, MS is associated with altered fecal bile acid and SCFA profiles; however, the cross-sectional design does not allow determination of whether these alterations preceded or followed the development of MS.

**Figure 3:**
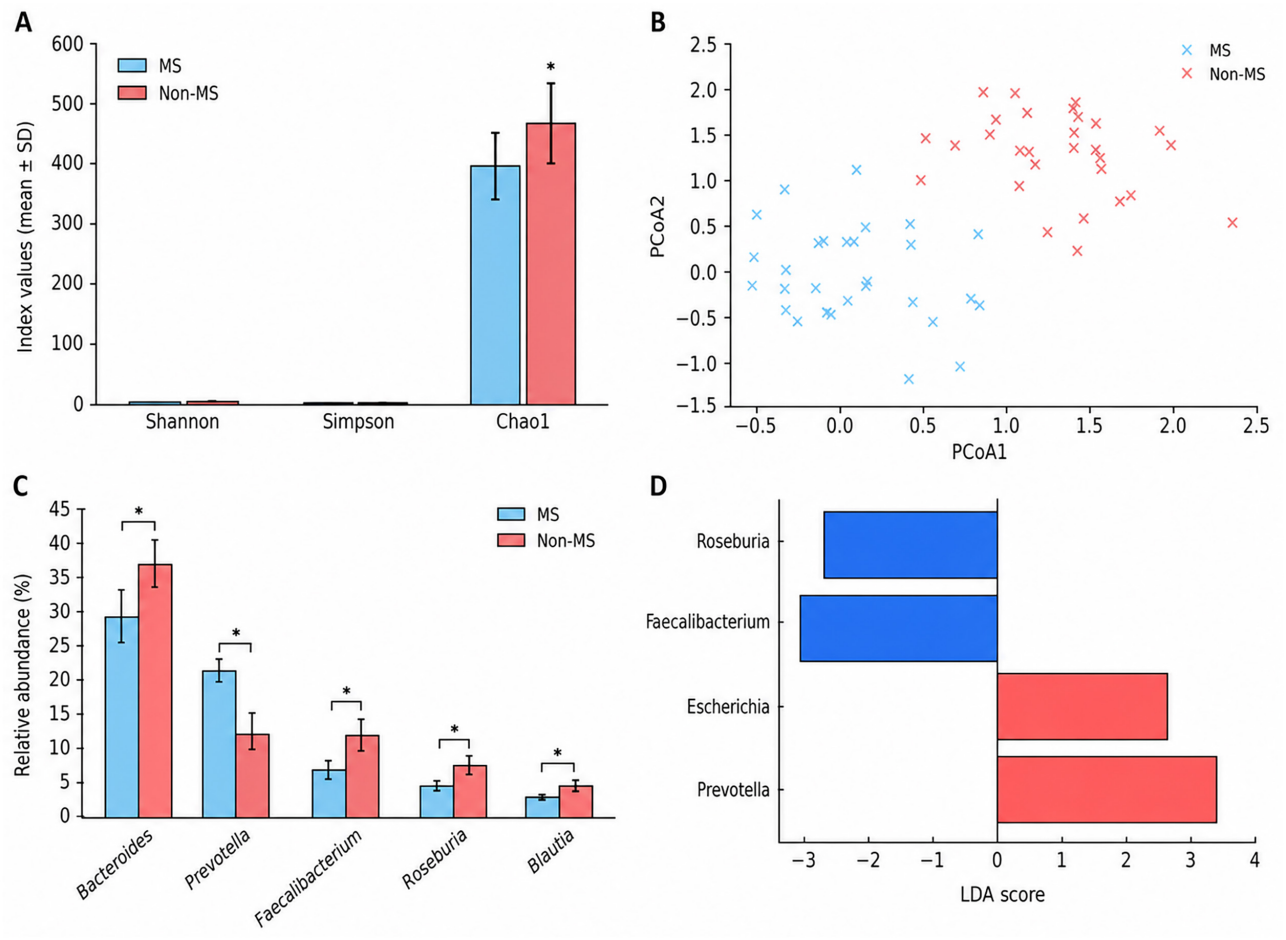
Gut microbiota diversity and differential bacterial taxa in post-cholecystectomy patients with and without metabolic syndrome. **(A)** Alpha-diversity indices, including Shannon, Simpson, and Chao1 richness indices, were compared between the MS and non-MS groups. **(B)** Principal coordinate analysis (PCoA) plot showing beta-diversity differences in fecal microbiota composition between the MS and non-MS groups. Each point represents one fecal sample. **(C)** Relative abundance of representative bacterial genera between the MS and non-MS groups. **(D)** Linear discriminant analysis effect size (LEfSe) showing discriminative bacterial taxa between groups. Positive LDA scores indicate taxa enriched in the MS group, whereas negative LDA scores indicate taxa enriched in the non-MS group. MS, metabolic syndrome; PCoA, principal coordinate analysis; LDA, linear discriminant analysis. *p < 0.05, **p < 0.01, ns, not significant.

Microbiota analyses revealed differences in gut ecosystem structure between groups. Among alpha-diversity indices, Chao1 richness was significantly lower in MS patients, whereas Shannon and Simpson indices were numerically lower but did not reach statistical significance (Figure 3A; Table 3). These findings suggest reduced microbial richness rather than a consistent decrease in overall alpha diversity or evenness. Beta-diversity analysis using principal coordinate analysis (PCoA) demonstrated separation between MS and non-MS groups (Figure 3B), indicating that overall microbial community structure differed according to MS status among post-CCE patients. Taxonomic comparisons showed increased Prevotella abundance and reduced Bacteroides, Faecalibacterium, and Roseburia abundance in the MS group (Figures 3C, D; Table 3). Blautia showed a decreasing trend in the MS group but did not reach statistical significance. The Firmicutes/Bacteroidetes ratio was lower in MS patients, reflecting compositional imbalance. Because Faecalibacterium and Roseburia are major butyrate-producing bacteria, their reduction was consistent with the lower fecal butyrate levels observed in the MS group.

**Table 3:** Gut microbiota diversity, composition, and bile acid/SCFA profiles in post-CCE patients with and without MS.

Parameter	MS group (*n* = 256)	Non-MS group (*n* = 176)	*p*-value
Shannon index	3.42 ± 1.34	3.89 ± 0.81	0.54
Simpson index	0.78 ± 0.30	0.84 ± 0.29	0.23
Chao1 richness	402 ± 50.0	478 ± 43.4	<0.01
Firmicutes/Bacteroidetes ratio	0.92 ± 0.25	1.48 ± 0.33	<0.001
Bacteroides (%)	29.30 ± 3.56	35.60 ± 3.91	<0.05
Prevotella (%)	21.60 ± 3.66	12.30 ± 3.00	<0.01
Faecalibacterium (%)	6.80 ± 1.26	12.10 ± 1.49	<0.01
Roseburia (%)	4.50 ± 0.81	7.80 ± 0.91	<0.05
Blautia (%)	3.10 ± 1.61	4.80 ± 0.92	0.03
Acetate (mmol/kg)	32.40 ± 4.56	40.70 ± 5.07	<0.001
Propionate (mmol/kg)	12.60 ± 2.21	16.20 ± 2.57	<0.01
Butyrate (mmol/kg)	7.20 ± 1.29	11.60 ± 1.77	<0.001
Valerate (mmol/kg)	2.10 ± 0.44	2.90 ± 0.50	0.06
Isobutyrate (mmol/kg)	1.30 ± 0.47	1.90 ± 0.35	0.09
Isovalerate (mmol/kg)	1.00 ± 0.24	1.60 ± 0.30	0.07
CA (μmol/g)	4.30 ± 0.56	3.10 ± 0.61	<0.05
CDCA (μmol/g)	3.20 ± 1.53	2.60 ± 1.27	0.12
DCA (μmol/g)	9.80 ± 1.03	5.40 ± 0.98	<0.001
LCA (μmol/g)	4.70 ± 0.73	2.20 ± 0.51	<0.001
UDCA (μmol/g)	2.10 ± 0.91	2.80 ± 0.85	0.18

### Fecal metabolomics and correlation analysis

**Figure 4:**
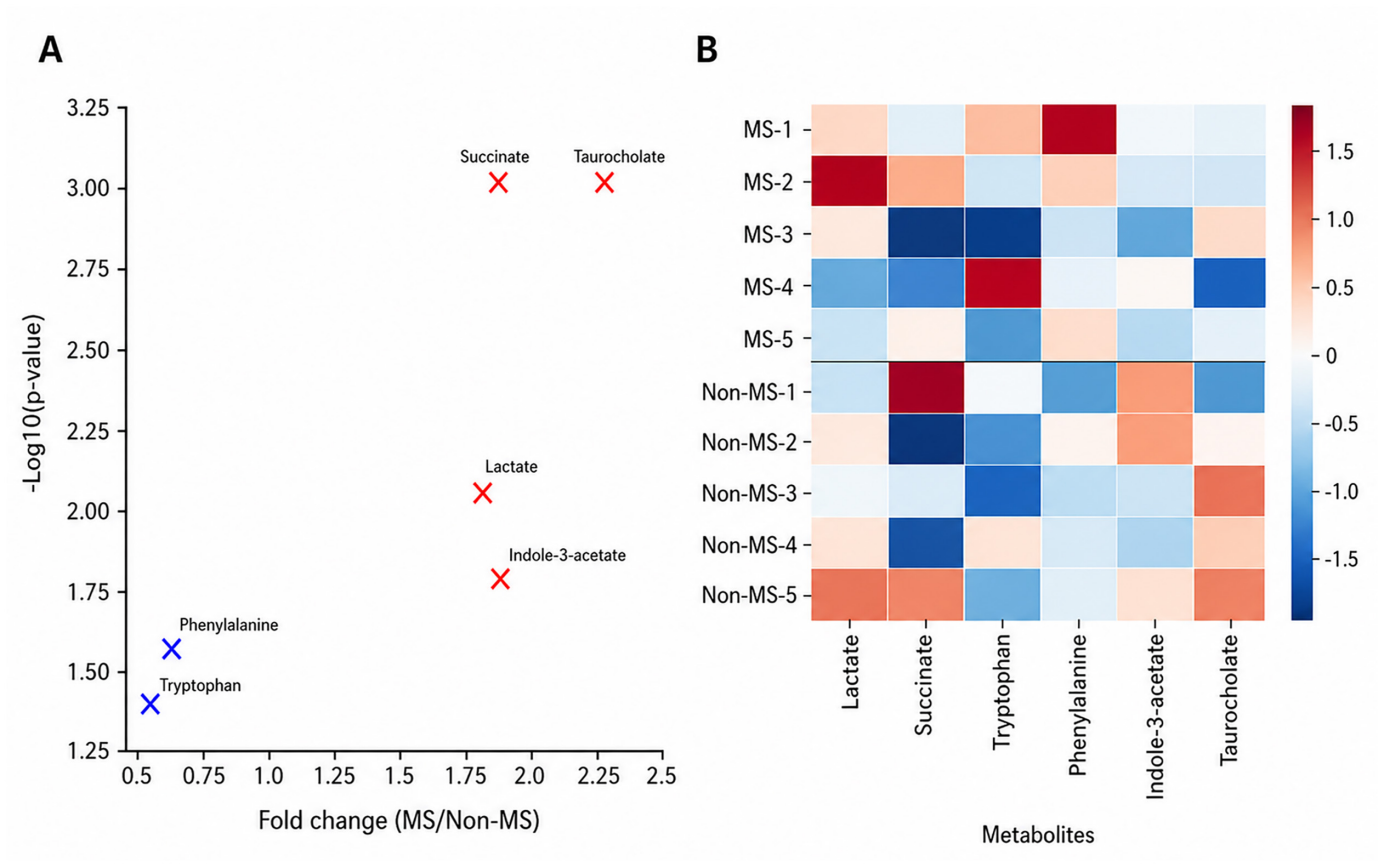
Differential fecal metabolomic profiles between post-CCE patients with and without MS. **(A)** Volcano plot showing differential fecal metabolites between the MS and non-MS groups. Red points indicate metabolites increased in the MS group, blue points indicate metabolites decreased in the MS group, and labeled metabolites represent major differential features. **(B)** Heatmap showing the relative abundance patterns of selected differential metabolites across 10 representative fecal samples, including five MS samples and five non-MS samples. CCE, cholecystectomy; MS, metabolic syndrome.

Untargeted fecal metabolomics provided additional insights into functional changes in gut metabolism (Figure 4; Table 4). Several metabolites were differentially abundant between MS and non-MS patients. Lactate, succinate, indole-3-acetate, and taurocholate were enriched in MS, whereas tryptophan and phenylalanine were depleted. Taurocholate, a taurine-conjugated bile acid, was particularly elevated, consistent with enhanced bile acid dysregulation. Multivariate analysis identified taurocholate and succinate as top discriminatory metabolites with high variable importance in projection (VIP) scores, indicating strong contributions to group separation. The volcano plot (Figure 4A) highlighted the main differential metabolites identified in the untargeted metabolomic analysis, and the heatmap (Figure 4B) visualized the relative abundance patterns of selected differential metabolites across 10 representative fecal samples, including five MS samples and five non-MS samples.

**Table 4:** Differential fecal metabolites between post-CCE patients with and without MS.

Metabolite	Fold change (MS vs. Non-MS)	VIP score	*p*-value
Lactate	1.8	1.92	<0.01
Succinate	2.1	2.34	<0.001
Tryptophan	0.6	1.45	<0.05
Phenylalanine	0.7	1.56	<0.05
Indole-3-acetate	1.9	2.12	<0.01
Taurocholate	2.3	2.46	<0.001

Correlation analysis integrated microbiota, metabolites, bile acids, and host parameters. *Prevotella* abundance correlated positively with triglycerides (*r* = 0.42, *p* < 0.001), supporting its role in lipid dysregulation. Faecalibacterium correlated negatively with HDL-C (*r* = −0.38, *p* < 0.01), although the biological interpretation of this association requires caution. Butyrate levels correlated inversely with HOMA-IR (*r* = −0.41, *p* < 0.01), linking microbial metabolites to systemic insulin resistance. Elevated DCA correlated positively with CRP levels (*r* = 0.47, *p* < 0.001), suggesting an association between altered bile acid metabolism and systemic inflammation.

### Multivariable-adjusted associations between MS status and microbiota/metabolomic features

To further evaluate whether the observed microbiota and metabolomic differences were independent of major clinical confounders, univariate and multivariable regression analyses were performed. In univariate models, MS status was significantly associated with lower Chao1 richness, lower Firmicutes/Bacteroidetes ratio, reduced Bacteroides, Faecalibacterium, and Roseburia abundance, decreased acetate, propionate, and butyrate levels, and increased CA, DCA, LCA, and selected differential metabolites. After adjustment for age, sex, BMI, smoking status, alcohol use, dietary pattern, medication use, and time since cholecystectomy, these associations remained largely unchanged. MS status remained independently associated with lower Chao1 richness, lower Firmicutes/Bacteroidetes ratio, reduced Bacteroides, Faecalibacterium, and Roseburia abundance, decreased acetate, propionate, and butyrate levels, and higher Prevotella, CA, DCA, LCA, lactate, succinate, indole-3-acetate, and taurocholate. These adjusted associations remained statistically significant after false discovery rate correction (Table 5).

**Table 5:** Univariate and multivariable-adjusted associations between MS status and key microbiota/metabolomic features in post-CCE patients.

Outcome	Univariate β (95% CI)	Univariate *p* value	Univariate FDR q value	Multivariable-adjusted β (95% CI)	Adjusted *p* value	Adjusted FDR *q* value
Chao1 richness	−76.00 (−85.13 to −66.87)	<0.001	<0.001	−77.64 (−88.64 to −66.64)	<0.001	<0.001
Firmicutes/Bacteroidetes ratio	−0.560 (−0.614 to −0.505)	<0.001	<0.001	−0.540 (−0.606 to −0.474)	<0.001	<0.001
Bacteroides (%)	−6.300 (−7.014 to −5.586)	<0.001	<0.001	−6.403 (−7.262 to −5.544)	<0.001	<0.001
Prevotella (%)	9.300 (8.644 to 9.956)	<0.001	<0.001	9.42 (8.62 to 10.21)	<0.001	<0.001
Faecalibacterium (%)	−5.300 (−5.562 to −5.038)	<0.001	<0.001	−5.347 (−5.665 to −5.029)	<0.001	<0.001
Roseburia (%)	−3.300 (−3.464 to −3.136)	<0.001	<0.001	−3.306 (−3.504 to −3.109)	<0.001	<0.001
Acetate (mmol/kg)	−8.299 (−9.218 to −7.381)	<0.001	<0.001	−8.499 (−9.607 to −7.390)	<0.001	<0.001
Propionate (mmol/kg)	−3.601 (−4.055 to −3.146)	<0.001	<0.001	−3.379 (−3.923 to −2.836)	<0.001	<0.001
Butyrate (mmol/kg)	−4.399 (−4.689 to −4.110)	<0.001	<0.001	−4.365 (−4.715 to −4.014)	<0.001	<0.001
CA (μmol/g)	1.200 (1.088 to 1.313)	<0.001	<0.001	1.213 (1.077 to 1.348)	<0.001	<0.001
DCA (μmol/g)	4.400 (4.205 to 4.594)	<0.001	<0.001	4.407 (4.172 to 4.642)	<0.001	<0.001
LCA (μmol/g)	2.500 (2.375 to 2.625)	<0.001	<0.001	2.545 (2.395 to 2.695)	<0.001	<0.001
Lactate	0.800 (0.741 to 0.860)	<0.001	<0.001	0.818 (0.746 to 0.890)	<0.001	<0.001
Succinate	1.100 (1.029 to 1.172)	<0.001	<0.001	1.081 (0.994 to 1.167)	<0.001	<0.001
Tryptophan	−0.400 (−0.432 to −0.367)	<0.001	<0.001	−0.392 (−0.431 to −0.353)	<0.001	<0.001
Phenylalanine	−0.300 (−0.331 to −0.268)	<0.001	<0.001	−0.295 (−0.332 to −0.257)	<0.001	<0.001
Indole-3-acetate	0.900 (0.833 to 0.967)	<0.001	<0.001	0.934 (0.853 to 1.014)	<0.001	<0.001
Taurocholate	1.301 (1.220 to 1.382)	<0.001	<0.001	1.270 (1.174 to 1.367)	<0.001	<0.001

## Discussion

The present study compared postoperative gut microbiota and metabolomic profiles between patients with and without MS after CCE. The main findings were that post-CCE patients with MS showed reduced Chao1 richness, a lower Firmicutes/Bacteroidetes ratio, depletion of SCFA-producing taxa such as Faecalibacterium and Roseburia, increased Prevotella and Escherichia coli abundance, elevated secondary BAs, and reduced fecal SCFAs. These associations remained after multivariable adjustment, suggesting that MS status was independently associated with an unfavorable gut microbiota–metabolite profile in post-CCE patients. However, these findings should not be interpreted as evidence that CCE or MS directly caused these alterations.

Another important finding of this study was the reduction in SCFA-producing bacteria and fecal SCFA concentrations. SCFAs, particularly butyrate, are important microbial metabolites involved in epithelial energy metabolism, immune regulation, and metabolic homeostasis (17). The decreased abundance of Faecalibacterium and Roseburia, together with lower butyrate levels, suggests impaired microbial metabolic function in post-CCE patients with MS. This pattern is consistent with previous post-CCE studies reporting enrichment of Prevotella and Escherichia coli and depletion of SCFA-producing taxa (1). Although secondary BA-serotonin signaling and lipid absorption pathways have been proposed in previous studies (2, 18), these mechanisms were not directly tested in the present study.

Consistent with previous studies, our results support the presence of bile acid-related microbial and metabolic alterations in post-CCE populations. Li et al. (28) reported reduced microbial diversity and enrichment of Prevotella in patients with post-CCE diarrhea, while Ridlon et al. (7) described the bacterial transformation of primary BAs into secondary BAs such as DCA and LCA. The increased DCA and LCA levels in the MS group are consistent with previous evidence that BAs function as metabolic regulators and signaling molecules (6, 29). Diet-induced changes in BA composition may also reshape microbial ecology and promote inflammatio (13), but the present retrospective data cannot determine causal pathways. Previous studies also suggest that diet may modify gut microbiota and metabolomic profiles after CCE, although this factor could not be fully evaluated in the present study. Western-style dietary patterns can reshape gut microbiota, alter BA metabolism, and affect host metabolic inflammation (20, 30, 31). Post-CCE studies have also reported altered microbiota composition, including enrichment of bile-acid-resistant taxa and depletion of SCFA-producing bacteria (3, 4). In addition, microbiota-derived DCA has been linked to carcinogenic risk in experimental obesity models (32). These studies provide biological context for the present findings, but our data cannot separate the relative contributions of diet, MS status, and surgical history. Therefore, the dietary and mechanistic implications of this study should be considered hypothesis-generating. The observed depletion of SCFA-producing taxa, reduced fecal SCFAs, and altered BA profiles suggest that microbiota-targeted or BA-modulating strategies could be explored in future prospective trials (6, 17, 29–31). However, no dietary, probiotic, prebiotic, or pharmacological intervention was tested in the present study. Therefore, these potential strategies should be interpreted only as future research directions rather than therapeutic conclusions.

Several limitations in this study should be acknowledged. First, this study was based on retrospective cross-sectional postoperative sampling. Because preoperative gallbladder disease patients and paired longitudinal samples were unavailable, we could not determine whether the observed microbiota or metabolomic differences developed after CCE, preceded MS, or reflected pre-existing metabolic differences between groups. A controlled longitudinal design including preoperative gallbladder disease patients with and without MS would be required to determine whether CCE has differential microbiota or metabolomic effects according to MS status. Second, although multivariable regression models adjusted for age, sex, BMI, dietary pattern, medication use, and time since CCE, residual confounding remains possible because dietary and medication variables were retrospectively collected and may not fully capture exposure intensity, duration, or adherence. Third, PICRUSt2-based functional prediction provides indirect inference of microbial functional potential rather than direct validation. Future studies should use longitudinal, multi-omics, and interventional designs to validate these associations and clarify potential mechanisms.

In conclusion, among patients with a history of cholecystectomy, those with metabolic syndrome exhibited distinct gut microbiota and metabolomic profiles, characterized by reduced microbial richness, altered bile acid profiles, and depletion of short-chain fatty acid-producing taxa. These findings indicate associations between MS status and gut microbiota–metabolite features in a post-CCE population, but they do not establish that CCE exacerbates microbiota or metabolic dysregulation in MS. Larger prospective, multi-omics, and interventional studies are needed to validate these findings and clarify their clinical relevance.

## Data Availability

The raw data supporting the conclusions of this article will be made available by the authors, without undue reservation.
